# Slide laryngotracheopexy for idiopathic subglottic stenosis

**DOI:** 10.1007/s00405-025-09226-x

**Published:** 2025-02-01

**Authors:** Zoltán Tóbiás, Ádám Bach, László Szakács, Miklós Csanády, Andrea Ambrus, László Rovó

**Affiliations:** 1 Department of Otorhinolaryngology-Head and Neck Surgery, Albert Szent-Györgyi Faculty of Medicine, University of Szeged, Szeged, Hungary; 2 Tisza Lajos krt. 111, Szeged, 6721 Hungary

**Keywords:** Airway stenosis, Idiopathic subglottic stenosis, Slide laryngotracheopexy

## Abstract

**Objectives:**

Idiopathic subglottic stenosis (iSGS) is a rare fibroinflammatory disorder characterized by scar tissue formation in the subglottic and tracheal regions. This study evaluated the long-term outcomes of a novel, single-step surgical technique that redefines the glottic and subglottic airway using local tracheal grafts.

**Methods:**

Thirteen patients (2 male and 11 female) diagnosed with iSGS who underwent slide laryngotracheopexy were enrolled in this study. The diagnosis of iSGS was confirmed through endoscopic assessment, CT scanning, and autoimmune blood testing. Patients completed post-operative Voice Handicap Index (VHI), Quality of Life (QoL), and MD Anderson Dysphagia Inventory (MDADI) questionnaires, and spirometry assessments were conducted.

**Results:**

All patients were successfully extubated in the operating room following surgery. None of the patients required intensive care unit treatment. The average hospital stay was 14 days. A temporary tracheotomy was needed in one case because of excessive crusting. Adjuvant endolaryngeal laser surgery was performed in three cases. In one case, mitomycin-C therapy was administered to treat granulation. Post-operative quality of life (QoL) assessment, peak inspiratory flow (PIF), and scores from the MDADI and VHI questionnaires were 9.0 (± 2.2), 2.8 l/s (± 0.83), 95.6 (± 4.3), and 18.7 (± 13.4), respectively.

**Conclusion:**

Slide laryngotracheopexy was a safe and dependable technique for cases classified as Cotton-Myers II-IV grade iSGS. The use of a tracheal flap was advantageous to ensure optimal mucosal function. Slide laryngotracheopexy may be employed following multiple endolaryngeal interventions, whereas adjuvant CO_2_ laser surgery or mitomycin-c therapy may be considered in cases involving granulation tissue formation.

**Level of evidence:**

4.

**Study Design:**

Retrospective case series review.

## Introduction

Idiopathic subglottic stenosis (iSGS) is a rare fibroinflammatory disorder. It is characterized by subglottic and tracheal scar tissue formation and primarily afflicts middle-aged Caucasian women. With a circumferential scar formation specifically from the upper edge of the cricoid to the first cartilage of the trachea, iSGS causes severe dyspnea and stridor, even asphyxiation if left untreated. If the symptom offset is within the adolescent or young adult period, it is often misdiagnosed as COPD or asthma bronchiale [1, 2]. Currently, there is no conservative therapy that resolves or decelerates subglottic scar progression. Because it is a progressive inflammation and characterized by a constant narrowing of the subglottic space, nearly every case requires surgery, and sometimes urgent surgical intervention is needed, such as tracheostomy, once the symptoms progress to a level of severe stridor and dyspnea.

Because there is no standard treatment protocol, surgical solutions vary widely based on the skill of the surgical team and the technical infrastructure of the institution. In cases of short-segment, low-grade (Cotton-Myer I–II) stenoses endolaryngeal interventions, such as balloon dilation, CO_2_ laser surgery, and coblation may be sufficient; however, because there is a high chance of re-stenosis, multiple surgeries may be needed to sustain good breathing function. For high-grade (Cotton-Myer III–IV) and/or recurrent stenoses open neck surgery is required. For these procedures, partial or extended cricotracheal resection (CTR) is the preferred surgical solution, which may involve stent/T-tube implantation, rib cartilage grafting, or temporary tracheotomy, though these additional steps are not always necessary. Open neck surgeries have a higher risk of postoperative complications. Airway obstruction by crusting or edema, anastomosis dehiscence, and wound infection during the early postoperative period, are severe complications. The chance of complications is significantly higher for sequential interventions [3–7]. Moreover, iSGS has a high recurrence rate, and many patients require multiple interventions within a year of their initial surgery. Thus, the expectations for a reliable surgical solution that ensures appropriate airway, breathing, and swallowing function, with acceptable voice quality and a low re-stenosis rate without the necessity of permanent tracheostomy or stent implantation, are high [1]. 

The development of new CTR and laryngotracheoplasty techniques has increased with promising long-term results. With multiple surgical solutions available, post-operative objective and subjective assessment of laryngeal functions is needed to determine the optimal modality. Thus, we conducted a retrospective study following up on the surgical outcomes of slide laryngotracheopexy, which is a novel, single-step surgical approach that redefines the glottic and subglottic airway by applying a local tracheal graft and rules out the need for tracheostomy or long-term stent/ T-tube implantation in cases of iSGS [3, 8–10]. 

## Materials and methods

A retrospective case series review study has been conducted at a single center. Ethical approval for this study was obtained from the ETT-TUKEB Hungarian National Ethical Committee, serial number: BM/2508-3/2024.

### Patients

Patients over 18 years of age diagnosed with ISGS were enrolled in this study. Inclusion criteria included those with advanced subglottic stenosis of at least grade II according to the Cotton-Myers classification, accompanied by worsening dyspnea or the need for a tracheostomy, as well as patients who had an inadequate response to previous endolaryngeal or CTR interventions and were indicated for slide laryngotracheopexy.

After endoscopic confirmation of subglottic stenosis, the diagnosis of idiopathic subglottic stenosis was based on the exclusion of autoimmune, traumatic, and iatrogenic origin (particularly prolonged intubation). The Cotton-Myer grading system was used for stenosis classification. HRCT (High-Resolution Computer Tomography) was performed in all cases to determine the exact length of the stenosis. Previous interventions and history of gastroesophageal reflux disease (GERD) were recorded. Autoimmune etiology was excluded by autoimmune blood screening for Antineutrophil Cytoplasmic Antibodies (ANCA), such as proteinase-3 (PR3) and myeloperoxidase (MPO) antibodies. We also screened for IgG4 antibodies.

### Surgical technique

**Fig. 1:**
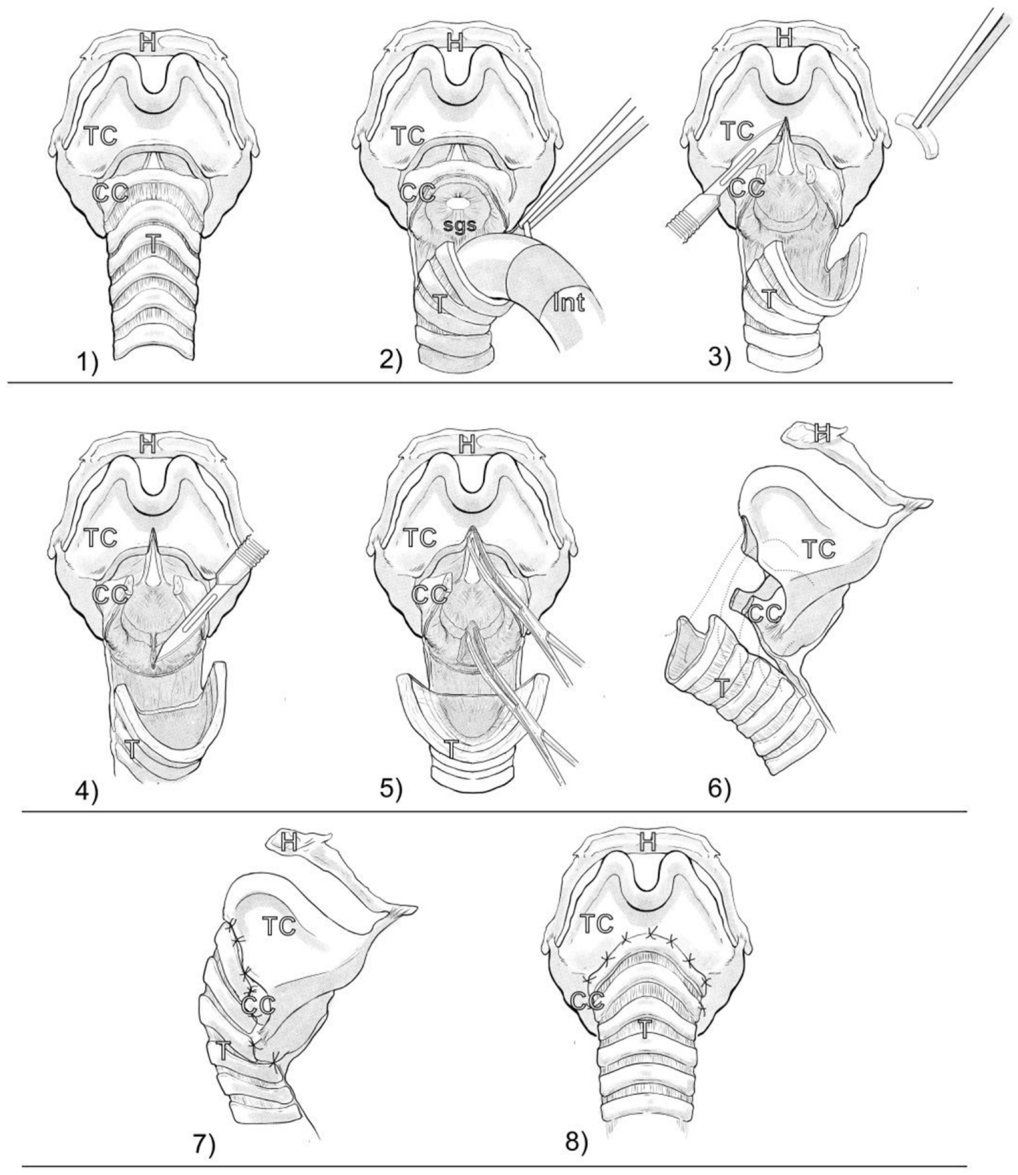
Schematic illustration of slide laryngotracheopexy. (1) Laryngotracheal complex is shown from below, ligaments are partially removed for better visual. (2) Dissection of the trachea (3) Excision of the cricoid arch and removal of scar tissue. Laryngofission is done below the level of the anterior commissure (4) posterior laminotomy of the cricoid (5) Enlarging subglottic space by expanding the fission and laminotomy by forceps (6) suture lines are shown with dashed lines (7) finished suture line of slide tracheoplasty (8) anterior view of slide laryngotracheopexy. Legend: H: hyoid bone; TC: thyroid cartilage; CC: cricoid cartilage; T: trachea; sgs: subglottic stenosis. Illustrations done by Zoltan Tobias MD

A detailed description of slide laryngotracheopexy was described in our previous publications. Therefore, only a summary of the surgical technique is reported here, with schematic illustrations presented in Fig. 1 [11]. 

The initial step of slide laryngotracheopexy was a horizontal incision on the neck at the level of the cricoid arch to expose the larynx and the trachea at the level of the cricotracheal junction. Next, a superior laryngeal release was performed by incision of the thyrohyoid membrane and the dissection of the superior cornu of the thyroid cartilage. The cricotracheal junction was separated with a circumferential dissection. Re-intubation was performed directly into the trachea, and an anterior midline laryngofission was performed with the preservation of the soft tissues of the anterior commissure. The arch of the cricoid was also transected and expanded, and a midline laminotomy was performed on the posterior plate. Following the laminotomy, the cricoid halves were bluntly dilated and separated using a dissector forceps. Thorough mobilization of the trachea was done by atraumatic dissection by sponge stick from the superior mediastinum. The pars membranacea was dissected from the esophagus to the level of the 3rd − 4th tracheal cartilage. The posterior wall was sufficiently cropped based on the size of the tracheal elevation and the tracheal trunk was pulled to the posterior mucosa of the cricoid. Before creating the anastomosis, a nasogastric tube was inserted.

In cases of previous PCTR surgery, the surgical approach remained consistent; however, specific adjustments were necessary in cases, where the anterior cricoid arch had been resected. The midline incision was made on the reconstructed anterior wall instead of the cricoid arch. Furthermore, as the trachea had been previously elevated, special attention was given to ensuring adequate mobilization and preserving the recurrent laryngeal nerves.

The anterior wall was reconstructed by the interpositioned tracheal cartilages between the extended lamina of the thyroid cartilage and cricoid. Thus, a wide and stable subglottic airway was created, in which the size of the lumen was significantly wider compared with the physiological one. During closure, two double-armed continuous locked sutures with knots placed on the external surface were initiated from the posterior midline and driven clockwise and counterclockwise until the threads met at the anterior midline, thus creating a continuous suture ring.

After closing the trachea, wound closure was performed following a standard neck incision, in multiple layers, with intracutaneous skin sutures. A Redon drain (12 CH) with a vacuum wound drainage system was placed in the midline. A cervical compression bandage was applied to minimize tissue disturbance in the pretracheal space during movement and to limit neck mobility.

### Post-operative care

During post-operative observation, a neck dressing was applied, and neck movements were strictly restricted. Intravenous meropenem therapy (or antibiotics depending on microbiological aspiration) was initiated before the operation and usually lasted for 10 days. Microbiological samples were collected intraoperatively. Neck drain removal occurred once the 24-hour output was less than 10 ml. Per os feeding began progressively after the 10th postoperative day. Every patient received intravenous proton-pump inhibitor (PPI) therapy (2 × 40 mg pantoprazole) during the hospital stay. The date of drain and nasogastric tube removal was registered.

### Functional and endoscopic evaluation

Evaluation of the postoperative results with respect to breathing, voicing, and swallowing was done on the 6th postoperative month. Peak Inspiratory Flow (PIF) was measured using a Spirotube spirometer (THOR Laboratories Inc, Székesfehérvár, Hungary). Voice Handicap Index and M.D. Anderson Dysphagia Inventory questionnaires were completed by the patients during follow-up visits. The functional outcomes of the surgery, including breathing, voice, swallowing, and overall satisfaction, were assessed using a quality of life (QOL) questionnaire [12]. Patients evaluated the following aspects using the specified scales: dyspnea (0 = absent to 4 = at rest), noisy breathing (0 = absent to 3 = very noisy even at rest), coughing (0 = absent to 2 = frequent episodes), dysphonia (0 = normal voice to 3 = aphonia), dysphagia (0 = absent to 3 = requiring nasogastric tube feeding), and global satisfaction (0 = outstanding to 4 = unsatisfied). A 70-degree rigid laryngoscopy was performed to assess the post-operative airway lumen. Additional imaging or direct laryngotracheoscopy was done only in cases of airway-related adverse events. For statistical analysis, JASP computer software (JASP Team 2023, Version 0.17. 3) was used.

## Results

Thirteen (2 male and 11 female) patients who were diagnosed with iSGS between December 2016 December and December 2022 were enrolled. The mean age was 59.3 ± 16.7 (min: 35, max: 82) years. For detailed patient information, see Tables 1 and 2. Seven patients had tracheostomy at the time of their primary assessment. Based on the HRCT scans, the stenosis longitudinal extension averaged 17.2 ± 7.2 mm. Patient data is listed in Table 2. Four patients had previous endolaryngeal laser surgery and/or balloon dilatation. In two cases, conventional PCTR (Partial Cricotracheal Resection) had already been performed at another institute; however, significant restenosis occurred.

**Table 1:** Detailed patient data (GERD: Gastro-esophageal reflux disease)

Patient	Sex	Age [years]	GERD	Smoking
1.	Female	82	No	Non-smoker
2.	Female	76	No	Non-smoker
3.	Male	33	No	Non-smoker
4.	Female	42	No	Non-smoker
5.	Female	76	Yes	Non-smoker
6.	Female	54	Yes	Non-smoker
7.	Male	76	No	Smoker
8.	Female	56	No	Non-smoker
9.	Female	35	No	Non-smoker
10.	Female	53	Yes	Non-smoker
11.	Female	50	No	Non-smoker
12.	Female	74	Yes	Non-smoker
13.	Female	64	Yes	Non-smoker

**Table 2:** Detailed patient data. F: female; M: male; CTR: cricotracheal resection

Patient/sex	Cotton-Myer grade	Stenosis length [mm]	Preoperative tracheotomy	Previous endolaryngeal interventions	Previous CTR
1 (F)	III	16.2	Yes	0	0
2 (F)	III	14.2	No	3	0
3 (M)	IV	17.0	Yes	0	0
4 (F)	II	16.5	No	0	0
5 (F)	II	14.9	Yes	0	0
6 (F)	III	6.0	No	1	1
7 (M)	III	25.0	Yes	0	0
8 (F)	III	20.0	No	0	0
9 (F)	II	22.5	No	0	0
10 (F)	III	10.0	No	10	0
11 (F)	III	8.3	Yes	0	1
12 (F)	III	33.0	No	0	0
13 (F)	III	19.8	No	2	0

All patients were successfully extubated in the operating room after surgery. None of the patients required Intensive Care Unit (ICU) treatment. The drain and nasogastric feeding tube, on average, were removed on the 5th and 11th postoperative day, respectively.

Dyspnea occurred from excessive crusting in patient #11 on the 5th postoperative day, which necessitated a temporary re-tracheotomy. Crusting was caused by difficult-to-expectorate airway secretion. Necrosis was not observed in the level of the anastomosis or in the tracheal mucosa. The patient was successfully decannulated after 5 days of observation and inhalation therapy. This patient had previous PCTR surgery. No other major complications were observed during the hospitalization of the remaining cases. The average length of hospitalization was 14 days.

In three cases, adjuvant CO_2_ laser vaporization was required because of granulation tissue formation after surgery within one year. In these cases, a tracheotomy was performed before surgery in another institute because of severe dyspnea. At the end of the first postoperative month, a mild granulation at the level of anastomosis had been observed in patient #6. Therefore, adjuvant topical mitomycin-c therapy was administered once. This patient also had PCTR surgery before inclusion.

**Fig. 2:**
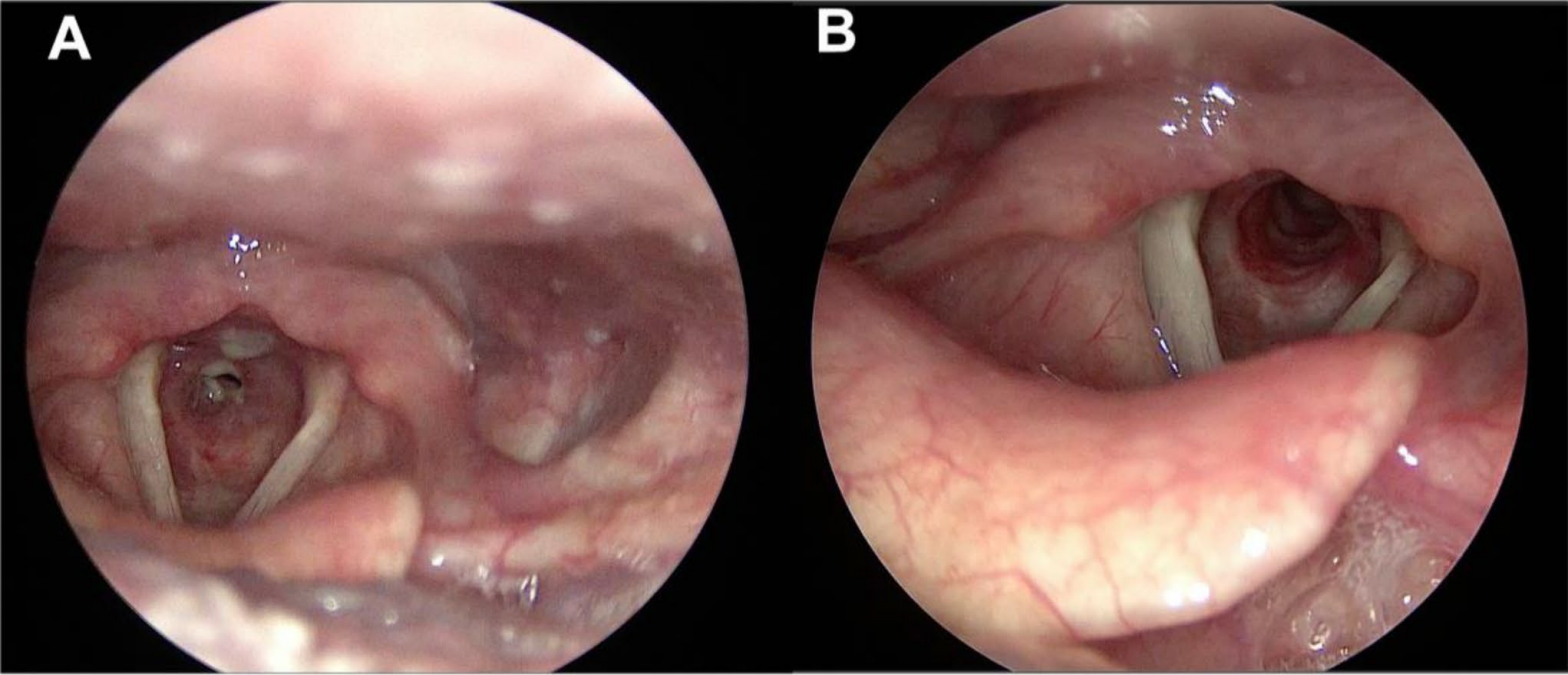
Endoscopic pictures of patient #13. **A**: preoperative Cotton-Myer grade III subglottic stenosis. **B**: Subglottic lumen in the 6th postoperative month

**Fig. 3:**
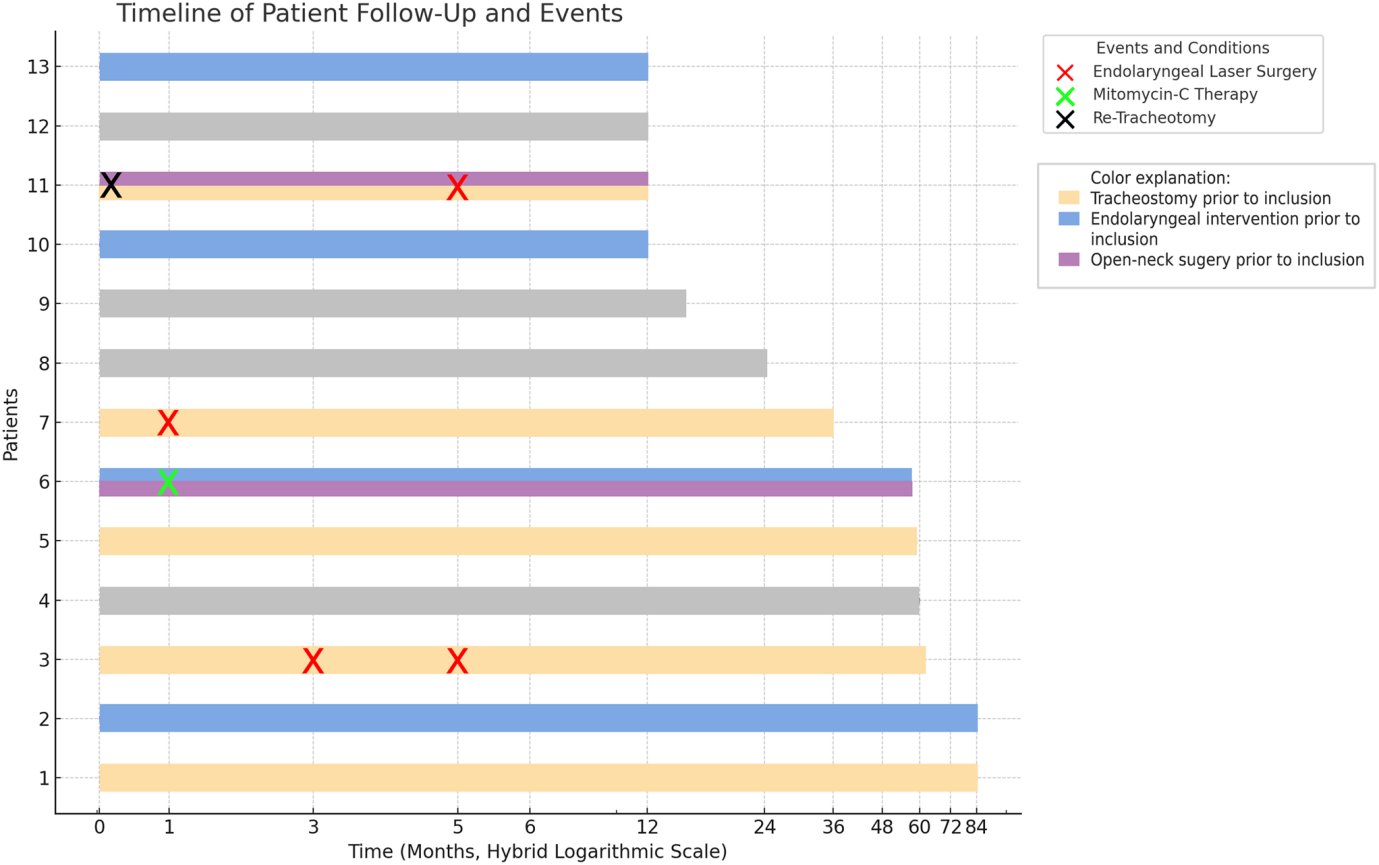
A timeline chart of patient follow-up

For the remaining cases, repeated and/or open neck surgery was not required. Adequate airway and breathing functionality were optimal during the follow-up time. We have not recorded any case of recurrent laryngeal nerve injury-related laryngeal paralysis. The PIF, QoL, VHI, and MDADI questionnaires, as well as the average scores, are also listed in Table 3. The minimum, maximum, and mean follow-up time was 12, 84, and 41 months, respectively. Pre- and postoperative endoscopic results are shown in Fig. 2. Conditions before inclusion and registered postoperative events are shown in Fig. 3.

**Table 3:** Postoperative functional results of surgery. QoL: quality of life; PIF: Peak Inspiratory Flow; MDADI: MD Anderson Dyphagia Inventory; VHI: Voice Handicap Index

	QoL	PIF [l/s]	MDADI	VHI
Mean	9.00	2.76	95.61	18.69
Std. Deviation	2.16	0.83	4.335	13.35
Minimum	6.00	1.85	86.21	7.00
Maximum	13.00	4.25	100.00	53.00

QoL - hSigher value represents better result; MDADI - value over > 80 indicates minimal to no impact on quality of life; VHI ≤ 10-15 indicates minimal to no perceived handicap.

After removing the nasogastric feeding tube, patients returned to an oral diet according to gradually built-up nutrition from pureed to a solid diet. All our patients remained decannulated with good breathing function according to the last follow-up.

## Discussion

Differential diagnosis of iSGS is done by exclusion of autoimmune, vascular, iatrogenic, traumatic, or malignant origin. Although c-ANCA antibodies may be present at some point during the disease progression, the autoimmune etiology is unclear. There is no evidence that iSGS is hereditary, yet remarkable cases of family connections have been reported. However, the general pathomechanism of scar tissue formation is well described, the initial trigger effect remains unknown in cases of iSGS. IL-23 and IL-17 A signal pathways initiate extracellular matrix formation and fibroblast hyperproliferation by upregulating TGF-β and MMP9. INF-γ expression is higher in cases of iSGS compared with cases of post-intubation stenosis. TGF-β was identified as one of the key factors in the underlying mechanism; however, there is no approved biological therapy. In this study, patients developed symptoms in their late adult years, whereas no juvenile asthma or COPD was mistakenly diagnosed [13, 14]. 

ISGS affects women more frequently. There are theories about the role of estrogen in abnormal inflammation and scar-forming; however, increased expression of estrogen and progesterone receptors has not been identified in cases of iSGS. The current hypothesis of iSGS etiology is the telescope phenomenon, which is observed during coughing movement. The contraction of tracheal muscles and the chronic mechanical friction result in the compressive disruption of the first tracheal ring, which results in venous congestion, consequent inflammation, and fibrosis of the subglottic area. During the coughing movement, the first tracheal ring may move inside the cricoid, thus causing mechanical injury. The cricoid mucosa is known for its sensibility and tendency for fibrosis. While the first ring of the trachea has supply capillaries running on the anterior surface of the cartilage, the cricoid has a dual-layered plexus of small and large vessels within and beneath the mucosa. Because of the telescope effect, the inner intramucosal capillaries are more affected by mechanical injuries; therefore, there is a higher chance of inflammation and fibrosis. Slide laryngotracheopexy redefines the laryngeal structure by bypassing the anterior part of the cricoid with a tracheal flap. This new cartilage framework may decrease the intensity and the affected area of the telescope effect, which may result in a lower recurrence rate. Patient history was examined for conditions that may cause chronic coughing. None of the patients were smokers and none had COPD-related symptoms. Extraesophageal reflux and the role of intramucosal pepsin absorption were examined as possible risk factors for iSGS. We searched the patient data for GERD history and most of our patients had episodes of severe reflux and ongoing therapy at the time of surgery [1, 2, 15–19]. 

There is no standard protocol for treating iSGS. Surgical solutions vary based on the surgeon’s skill and the level of care provided by the institution. Currently, balloon dilatation and CO_2_ laser scar removal surgery are the most common endolaryngeal procedures. Endolaryngeal surgery may be sufficient in patients with mild (Cotton-Myer I-II; shorter than 1 cm in cranio-caudal dimension) stenoses. The Maddern procedure is an innovative solution that yields promising results. An endolaryngeal shaver resection may be performed, then the mucosa is relined with buccal graft implantation, which is maintained by a silicon stent for two weeks [1, 4, 5, 7, 20]. 

Endolaryngeal procedures may be suitable for mild stenosis, but they are not applicable for Cotton-Myer III-IV grade stenoses, because of the high recurrence rate of iSGS that typically requires repeated interventions. Multiple endolaryngeal laser surgeries may cause hourglass malformation of the affected tracheal section because of the collateral thermocoagulation injury of the tissues. The recurrence rate is significantly lower following cricotracheal resection; however, the perioperative risk and voice outcome are inferior compared with endolaryngeal procedures. Most CTR participants require tracheostomy or have already been tracheotomized and have experienced multiple endolaryngeal procedures. In contrast, novel techniques involving cricotracheal resection improve quality of life and require shorter hospitalization. To prevent re-stenosis, a combined intervention of endolaryngeal/open-neck surgery with local adjuvant therapy, such as topical mitomycin-c, may be used. The demand for a reliable, successful open-neck surgery for iSGS with good functional results is high, because endolaryngeal procedures are often insufficient, and tracheotomy is associated with a poor quality of life, especially in young adult patients.

Cricoid cartilage serves as the fundament of the laryngeal structure. Surgery affecting the cricoid may subsequently cause swallowing, phonation, or breathing problems. During conventional PCTR, an end-to-end anastomosis is formed between the remnant of the cricoid arch, the trachea, and the lamina of the thyroid. This procedure may be further extended with an interpositioned rib cartilage graft and/or stent implant during ECTR. Laryngosternopexy may be used to decrease the risk of anastomosis dehiscence. An autologous graft is usually harvested from rib cartilage however, secondary wound infection at the donor site must be considered. Stent implantation may cause serious adverse events if crusting or severe granulation occurs. Even as a temporary solution, Montgomery T-tube implantation affects quality of life, and a subsequent intervention is required for removal [3, 8–10, 21]. 

During slide laryngotracheopexy, the anastomosis is formed in an elliptic manner, resulting in a wider subglottic space. This is achieved with an abundant extension of the cricoid arch and the plate without extensive resection of the cartilage to form a side-to-end, rather than end-to-end, anastomosis. Thus, even in a case of re-stenosis, the airway remains sufficiently wide for good breathing and phonation. The interpositioned tracheal flap is ideal for reconstruction because it contains airway mucosa with a rich blood supply; thus, wound healing and mucosal clearance will be optimal. However, the sensitive blood supply must remain intact, which requires careful preparation. The elliptic anastomosis line prevents horizontal rotation of the larynx. Therefore, there is a reduced chance of mechanical injury and dehiscence. Furthermore, a longer anastomosis perimeter can be established with more sutures to decrease the tension applied to a single suture.

Active arytenoid mobility and intact sensory innervation are key to preventing aspiration. During slide laryngotracheopexy, the cricoid plate is not resected; therefore, the arytenoids and interary region remain intact. Based on the MDADI questionnaire results, patients have good swallowing function following surgery. Furthermore, with the preservation of arytenoid mobility and vocal folds, optimal expectoration is assumed, even after modification of the laryngeal structure.

According to results in literature, there was no significant difference in the postoperative care period between slide laryngotracheopexy and conventional CTR surgery [22, 23]. In cases involving postoperative complications, patients had a tracheotomy and multiple endolaryngeal interventions before surgery, which altered regenerative capacity, blood supply, and the local microbiome. We believe that scar tissue formation and mucociliary dysfunction from the first open-neck surgery altered the local healing capacity, which led to an airway secretion-related severe complication. This is why we aim to find a permanent solution for ISGS that negates additional open-neck surgeries.

PIF and endoscopic measurements confirm the long-term stable subglottic space following surgery without significant restenosis. Based on the QoL and MDADI questionnaire results, the patients were satisfied with the long-term results of surgery. Recurrent nerve injury is unlikely because the cricoid is not extensively resected. The procedure preserves the anterior commissure and vocal cords and ensures good phonation. Based on the VHI questionnaire results, the patients are satisfied with the phonation quality.

## Limitations

The estimated prevalence of iSGS is 1:400,000. The limitations of this study are the small patient population and the single-center experience. No preoperative VHI and MDADI scores were recorded, since only 31% (4 patients) of the enrolled population had no record of previous intervention or tracheostomy at the time of inclusion. This study was conducted in a tertiary care unit, and patients were acquired from all over the country, even from abroad. A greater patient population with conventional and extended CTR surgery is planned for future studies. Our team plans to continue further evaluation using Fiberoptic Endoscopic Evaluation of Swallowing (FEES) to assess objective swallowing function parameters.

## Conclusion

This study evaluated the long-term outcomes of a novel open-neck surgical approach for idiopathic subglottic stenosis (iSGS). Slide laryngotracheopexy, a one-step open neck procedure, involves using a local tracheal flap as a graft to bypass the stenotic section, without resecting the cricoid or trachea. Based on our results, slide laryngotracheopexy is a secure and dependable technique for cases classified as Cotton-Myer II-IV grade iSGS. The use of a tracheal flap was advantageous in ensuring optimal mucosal function. Selecting the most suitable surgical method poses challenges because of the variations in stenosis severity and clinical outcomes. Slide laryngotracheopexy may be performed following multiple endolaryngeal interventions, whereas adjuvant CO_2_ laser surgery or mitomycin-c therapy may be considered in cases with granulation tissue formation. Building on a promising decade of experience, we intend to continue accumulating iSGS cases and promote the use of slide laryngotracheopexy as an innovative solution for stenosis surgery.
